# Novel Approaches for Concurrent Irradiation in Locally Advanced Cervical Cancer: Platinum Combinations, Non-Platinum-Containing Regimens, and Molecular Targeted Agents

**DOI:** 10.1155/2013/536765

**Published:** 2013-05-21

**Authors:** Giannis Mountzios, Aspasia Soultati, Dimitrios Pectasides, Meletios A. Dimopoulos, Christos A. Papadimitriou

**Affiliations:** ^1^Department of Medical Oncology and Translational Research, 251 Air Force General Hospital, Neo Psychiko, 154 51 Athens, Greece; ^2^Department of Clinical Therapeutics, University of Athens School of Medicine, “Alexandra” University Hospital, 115 25 Athens, Greece; ^3^Second Department of Internal Medicine, University of Athens School of Medicine, “Hippocrateion” Hospital, 115 25 Athens, Greece

## Abstract

Despite the available prevention and early detection strategies, squamous-cell carcinoma of the uterine cervix is still diagnosed as locally advanced disease in a considerable proportion of patients. As a potent sensitizer of cancer cells, cisplatin has been the “traditional partner” of external beam irradiation in this setting for more than two decades. Induction chemotherapy strategies followed by concurrent chemoradiation or surgery and preoperative concurrent chemoradiation have been recently implemented in clinical trials in an effort to optimize local control and to minimize the risk of distant metastases. In this context, cisplatin has been combined with a number of other potential radiosensitizers, including 5-fluorouracil, capecitabine, and gemcitabine. In patients resistant or intolerant to platinum compounds, numerous non-platinum-containing regimens have been developed, implementing various antimetabolites, taxanes, antineoplastic antibiotics, and topoisomerase II inhibitors. More recently, molecular agents targeting critical pathways in cervical malignant transformation are being assessed in early clinical trials in combination with external-beam irradiation. In the current work, we review the evolving role of cisplatin and other platinum compounds, either alone or in combination regimens, in the context of other potent radiosensitizers. The emerging role of molecular targeted agents, as candidate partners of external beam irradiation, is also discussed.

## 1. Introduction

Squamous carcinoma of the uterine cervix, often referred to as cervical cancer, remains a major concern for public health. Worldwide, cervical cancer accounted for 287,000 deaths in 2008, and the number is expected to rise up to 410,000 by 2030 [1, 2]. Despite the worldwide implementation of prevention and early detection strategies, including the Papanicolaou smear test, human papillomavirus (HPV) testing, and vaccines, approximately 30% of newly diagnosed cases still fall into the category of “locally advanced disease,” indicating tumor spreading outside the uterine cervix (*Féderation Internationale Gynécologie Obstétrique* (FIGO) stages IIA-IVA) or bulky disease confined in the uterine cervix (FIGO stage IB2), at the time of diagnosis [3]. Moreover, 50% of patients with locally advanced disease are expected to relapse within the first 2 years after initial treatment [4].

Cisplatin monotherapy, often combined with external-beam irradiation, remained the dominant treatment for locally advanced disease for more than fifteen years [5]. More recently, induction chemotherapy strategies followed by concurrent chemoradiation or surgery and preoperative concurrent chemoradiation have been implemented in the therapeutic armamentarium in an effort to optimize local control and at the same time to minimize the risk for metastatic disease. In this context, cisplatin has been combined with a number of other potential radiosensitizers to enhance cytotoxic activity. In parallel, numerous non-platinum-containing regimens have been developed for patients who either fail or become intolerant to platinum compounds. More recently, molecular agents targeting critical pathways in cervical malignant transformation are being assessed in early clinical trials in combination with external-beam irradiation, heralding the era of concurrent “bioradiotherapy” for locally advanced cervical cancer. In the current work, we review the evolving role of cisplatin and other platinum compounds, either alone or in combination regimens, in the context of other potent radiosensitizers. Moreover, we discuss the emerging role of molecular targeted agents as candidate partners of external beam irradiation in patients with locally advanced cervical cancer.

## 2. Concurrent Chemoradiation Based on Platinum-Containing Regimens

Radiation alone fails to control disease in over 35% of patients with cervical cancer diagnosed at FIGO stages IB2-IVA [6]. Five-year survival rates up to 72.2%, 63.7%, 41.7%, and 16.4% for stages IB2, IIB, IIIB, and IVA, respectively, have been reported with exclusive radiation [7]. Concurrent chemoradiation has led to a significant benefit in reducing both local and distant recurrences in five randomized studies [8–12] that involved a total of 1,894 women. In the trial conducted by the Radiotherapy Oncology Group (RTOG), Morris et al. [12] randomized 401 stage IB-IVA patients to either concurrent chemoradiation with cisplatin and 5-fluorouracil (5-FU) or to extended-field radiation alone (control group). Concurrent chemoradiotherapy resulted in a 5-year overall survival rate of 73% compared to 58% for radiation alone and decreased the rates for both local and distant recurrences. In another prospective phase III multicenter randomized trial reported at the same time with the former one, Rose et al. [11] recruited 526 evaluable patients with stage IIB-IVA cervical cancer in a Gynecologic Oncology Group (GOG) three-arm trial that compared weekly cisplatin versus cisplatin and 5-FU and hydroxyurea versus hydroxyurea alone, concurrently with radiation therapy. Superior survival rates for both cisplatin-containing regimens (66% and 64%, resp.) compared with hydroxyurea alone (39%) were reported [11]. Moreover, cisplatin monotherapy was proven to be less toxic than the cisplatin/5-FU/hydroxyurea combination [11] or the protracted venous infusion (PVI) 5-FU [13]. Whitney et al. [10] randomized 388 patients with stage IIB-IVA disease in a Gynecologic Oncology Group (GOG) trial to receive either radiation therapy with concurrent cisplatin and 5-FU or hydroxyurea. Patients in the cisplatin arm had a significantly better 5-year survival rate (63% versus 47%). In a meta-analysis based on 19 trials (including a total of 4,580 patients), an absolute survival benefit of 12% at 5 years with concurrent chemoradiation based on cisplatin as compared to radiation alone was demonstrated [14]. An update of the same work comprising 24 trials involving 4,921 patients showed that chemoradiation improves both overall survival (OS) and progression-free survival (PFS), when a platinum compound was used, with an absolute benefit of 10% [15]. Importantly, in a pilot study conducted by Nugent et al. [16], the number of cisplatin chemotherapy cycles was independently predictive for PFS and OS: patients who received less than six cycles had worse clinical outcome as compared to those who completed at least six cycles of treatment. Regarding the optimal dosing, a randomized trial comparing cisplatin at 40 mg/m^2^ weekly with cisplatin at 75 mg/m^2^ every 3 weeks reported twice as many delays of therapy with the higher, less frequent cisplatin administration [17]. On the ground of available evidence regarding clinical efficacy and acceptable tolerance, weekly cisplatin at the dose of 40 mg/m^2^ is considered the standard regimen that other agents should be compared to [18].

Although carboplatin may also serve as an active radiosensitizer [19–23] and is less toxic than cisplatin in patients with renal dysfunction due to ureteral obstruction, efficacy results from phase I-II trials (Table 2) are generally modest and objective response rates (ORR) are inferior to those reported with cisplatin [20–24]. Multiple combinations that incorporated carboplatin have been evaluated, with the combination of docetaxel with carboplatin exhibiting encouraging results [21, 22]. The weekly paclitaxel and carboplatin chemoradiation regimen has been also proven feasible and active in phase I trials [23, 24], yet dose-limiting diarrhea makes this regimen poorly tolerated [25]. Concurrent chemoradiation with tegafur-uracil (UFT) and carboplatin showed no difference in respect to ORR, PFS, OS, and treatment-related toxicity as compared to carboplatin alone in a prospective, phase III trial that recruited 469 patients with stage IIB-IIIB cervical cancer [26]. Finally, in a case-control study [27], the combination of radiotherapy, concurrently with 5-FU and carboplatin, has been compared to radiation alone in the same setting: the authors reported similar ORR, DFS, and OS between the two groups of patients. Acute toxicity, primarily hematologic, was significantly higher in the cases than in the controls (25% versus 3%) [27].

Since 5-FU represents a potent radiosensitizer too, a number of studies have been undertaken to clarify the use of both agents in combination with pelvic radiotherapy. In a study by Kim et al. [28], 158 patients with FIGO stage IIB through IVA disease were assigned to either monthly 5-FU and cisplatin or weekly cisplatin concurrent with pelvic radiotherapy and high-dose rate brachytherapy. The response rate for each group was 91%. Four-year OS and PFS rates were 70% and 67%, respectively, with the combination regimen, while with weekly cisplatin they were 67% and 66%, respectively. The authors concluded that chemoradiation with weekly cisplatin significantly improved compliance with treatment and reduced acute hematologic toxicity without affecting response and survival rates compared to the combination arm. Another trial designed to compare protracted venous infusion (PVI) of 5-FU with standard weekly cisplatin and concurrent RT in patients with stages IIB, IIIB, and IVA cervical cancer was prematurely terminated when a planned interim analysis indicated that the PVI 5-FU/RT treatment arm had a higher treatment failure rate (35% higher) and would, most likely, not result in any improvement in PFS compared with weekly cisplatin/RT [13]. Yet clinical interest in 5-FU and its combinations is still active: recently, in a study that reevaluated the efficacy of concurrent chemoradiation using 5-FU and cisplatin in 57 patients with stage IIB-IVA and bulky IB2-IIA tumors, an ORR of 91.5% with a 5-year OS and a 3-year PFS rate of 69.4% and 74.9%, respectively, was reported [29]. In the same context, another study [30] compared survival outcomes and toxicities between concurrent chemoradiotherapy with cisplatin plus 5-FU and cisplatin plus paclitaxel in 93 patients with locally advanced cervical carcinoma: no significant differences were found in 5-year PFS or OS between the two treatment groups. Nevertheless, the cisplatin plus paclitaxel arm was associated with increased leukopenia, neutropenia, and peripheral neuropathy but less gastrointestinal toxicity (nausea) compared to the cisplatin plus 5-FU arm [30].

Capecitabine, an oral prodrug of 5-FU, has been also evaluated in the same setting with promising results. In a phase I study of daily capecitabine combined with weekly cisplatin and radiotherapy, a PFS at 12 months of 69.2% and at 24 months of 49.2% with an OS rate of 57.7% at 24 months was reported [31]. Following these results, a phase II study [32] evaluated 60 patients with stage IIB-IIIB disease who received capecitabine during radiation, followed by six cycles of capecitabine monotherapy. The ORR was 88%, while the 1-year PFS and OS rates were 86% and 95%, respectively. At 23 months, 76% of patients were progression-free and complete response (CR) was maintained in 90% of the 48 patients who originally achieved a CR [32].

In a pilot phase II study designed to investigate the feasibility, efficacy, and safety of gemcitabine in combination with irradiation in 19 chemonaïve patients with FIGO stage IIIB cervical cancer, a CR was observed among 17 (89.5%) of them and after a median follow-up time of 19.9 months, all patients were alive with sixteen of them remaining relapse-free [47]. On these grounds, the gemcitabine-cisplatin combination was administered concurrently with radiotherapy in a phase I/II study resulting in a 97.3% ORR (88.8% were complete responses). The 3-year RFS and OS rates were estimated to be 67% and 72%, respectively [34]. These encouraging results led to a large randomized, phase III trial [33] (Table 1) designed to determine whether the addition of gemcitabine to concurrent cisplatin chemoradiotherapy could improve outcome compared with current standard of care in locally advanced cervical cancer: five hundred and fifteen patients with stage IIB-IVA disease were randomly assigned to either cisplatin and gemcitabine, weekly for 6 weeks with concurrent external-beam radiotherapy, followed by brachytherapy and then two adjuvant cycles of cisplatin plus gemcitabine, (arm A) or to cisplatin and concurrent radiotherapy followed by brachytherapy only at the same doses (arm B). PFS at 3 years was significantly improved in arm A versus arm B (74.4% versus 65.0%, resp.; *P* = 0.029) as were overall PFS (hazard ratio (HR) = 0.68; 95% CI, 0.49 to 0.95; *P* = 0.0227) and OS (HR = 0.68; 95% CI, 0.49 to 0.95; *P* = 0.0224). The authors concluded that gemcitabine plus cisplatin chemoradiotherapy followed by brachytherapy and adjuvant gemcitabine/cisplatin chemotherapy improved survival outcomes with increased but clinically manageable toxicity when compared with standard treatment [33]. Although these results challenge the current standard of cisplatin monotherapy, since patients in the experimental arm also received two cycles of adjuvant chemotherapy, it is not clear to what extent the survival benefit observed in the experimental arm is attributable to the addition of gemcitabine in the chemoradiation phase or to the addition of an adjuvant chemotherapy phase itself.

Nedaplatin is a synthetic analog of cisplatin that exhibits less nephrotoxicity, neurotoxicity, and gastrointestinal toxicity. Weekly nedaplatin concurrently with radiation achieved an ORR of 90%, a 3-year PFS of 58.7%, and an OS of 78.0% in a pilot phase II trial [48]. In a following randomized phase II study, nedaplatin-based concurrent chemoradiotherapy showed superior clinical efficacy and no statistically significant difference in toxicity as compared to radiotherapy alone [49]. Multiple combinations incorporating nedaplatin have also been evaluated, mainly in phase I trials. The combination of paclitaxel and nedaplatin concurrently with radiotherapy, followed by consolidation treatment with the same regimen, resulted in a CR of 88% and an estimated 2-year PFS and OS rate of 82% and 93%, respectively [35].

## 3. Concurrent Chemoradiation Based on Non-Platinum-Containing Regimens

Among taxanes and other microtubule-targeting agents, paclitaxel has been extensively evaluated within chemoradiation regimens. The feasibility of concurrent radiotherapy and paclitaxel administration was evaluated in a pilot study with 20 patients (13 new cases, stage IIB-III, and 7 with pelvic recurrences) and complete regression was reported in 63% [50]. In a subsequent randomized phase II trial, weekly cisplatin was compared to weekly paclitaxel as concurrent chemotherapy with standard RT in patients with stage IB2-IVA disease or with postsurgical pelvic recurrence: the proportion of patients surviving at 2 and 5 years was 78% and 54% for the cisplatin arm and 73% and 43% for the paclitaxel arm, respectively, thus suggesting that weekly paclitaxel does not provide any clinical advantage over weekly cisplatin [36]. A multiagent regimen that included paclitaxel, ifosfamide, and cisplatin (TIP) has been also evaluated in two different settings: bulky and locally advanced cervical cancer and recurrent-persistent disease in a total of 38 patients—eleven women achieved a clinical CR, 21 had a partial response, and only one patient had progressive disease (PD), accounting for an impressive ORR of 84.2% [37]. Finally, the combination of paclitaxel and vinorelbine was associated with significant hematologic toxicity [51].

Mitomycin C is an antineoplastic antibiotic drug that has been extensively evaluated in the locally advanced cervical cancer setting: in an earlier study, 40 patients with stage IB-IVA disease received mitomycin C and 5-FU followed by sequential pelvic irradiation—a complete response rate of 63%, a local control rate of 58%, and a 5-year survival rate of 44% were obtained which were not superior to those achieved with radiation alone [52]. Another larger trial randomized 160 patients with locally advanced disease to receive either RT alone or RT with concomitant mitomycin C: the four-year DFS rates for RT with mitomycin C and RT alone were significantly different (71% versus 44%) [38]. Using a combination regimen of 5-FU with mitomycin C and radiotherapy, Ludgate et al. suggested an improvement in pelvic control and in the 3-year survival rate for the combined modality compared to RT alone (55% versus 28%) using, however, historical controls with remarkably low response rates as a reference [53]. Similar results were obtained in another study using the same combination concurrently with RT, although the authors commented that the regimen failed to control distant metastasis in late-stage patients [39]. In a pilot trial, 60 women with advanced cervical cancer were treated with a combination of external and intracavitary RT along with one cycle of 5-FU and mitomycin C and a second cycle of 5-FU and cisplatin. The 5-year OS for stage IIB and IIIA-IVA patients was 48% and 39%, respectively [54]. Christie et al. reviewed 177 patients treated with pelvic radiotherapy for locally advanced disease and focused on 93 patients who had received chemotherapy with infusional 5-FU with or without bolus mitomycin C. The median OS for all patients was 47 months but was significantly higher (87 months) for the combination regimen group. Rates of PFS and local control were also higher in the same group, albeit at the cost of substantial toxicity, with 36% of patients in the combination arm experiencing grade 3 or 4 complications [40]. Finally, in a phase II trial [41], women with FIGO stage IIB-IVA disease who received cisplatin, 5-FU, mitomycin C, and concomitant radiotherapy achieved an ORR of 82%. All patients developed acute hematological toxicity and two patients experienced severe late bowel toxicity. The lack of clinical efficacy improvement compared to historical controls treated with cisplatin alone and the late bowel toxicity discouraged further use of that regimen [41].

Topotecan and irinotecan are topoisomerase II inhibitors that have been evaluated in combination with RT in locally advanced cervical cancer. The feasibility of adding weekly topotecan to cisplatin in 12 patients with stage IB2-IVA disease receiving pelvic irradiation has been affirmed in a pilot trial and responses up to 92% were reported [55]. In a validation phase I cohort, intravenous topotecan at the dose of 0.5 mg/m^2^ and cisplatin at the dose of 30 mg/m^2^ given weekly for 6 weeks with concurrent pelvic radiation and intracavitary brachytherapy were tolerable [42]. The safety and feasibility of concurrent radiation therapy and weekly irinotecan in patients with locally advanced disease were also reported in two small phase II trials, showing promising efficacy and mostly tolerable adverse events [43, 56]. However, further and larger studies will be required in order to clarify the exact role of camptothecins in combination with RT in the locally advanced setting of cervical cancer.

## 4. Molecular Targeted Agents as Radiosensitizers

The ongoing elucidation of the critical steps in the process of malignant transformation of the cervical epithelium has revealed important biological pathways, including neoangiogenesis and proliferate signal transduction, as appealing therapeutic targets for molecules targeting these aberrant pathways. Consequently, a number of biological agents are currently in clinical development (Table 3), aiming at inhibiting angiogenesis, targeting epidermal growth factor receptor (EGFR), cell cycle, histone deacetylases, cyclooxygenase-2 (COX-2), or mammalian target of rapamycin (mTOR) [57]. In locally advanced disease, in particular, the potential synergy between the cytotoxic effect of irradiation and the proapoptotic effect of molecular agents targeting pathways crucial for cancer cell survival has generated the hypothesis that administration of these agents concurrently with radiotherapy may enhance clinical benefit while reducing chemotherapy-associated toxicity [58]. EGFR-targeting agents were the first molecules to be tested as radiotherapy partners in this setting, with the EGFR tyrosine kinase inhibitor erlotinib demonstrating feasibility and safety in concomitant administration with radiotherapy at the dose of 150 mg daily in a pilot phase I trial [44]. The chimeric monoclonal antibody against EGFR cetuximab was also tested in combination with radiotherapy in patients with newly diagnosed locally advanced cervical cancer in a recently reported phase I trial showing that the combination was feasible for patients receiving pelvic radiotherapy but not feasible for patients receiving extended field radion therapy due to nodal metastases [45]. Based on this preliminary evidence, a phase II pilot trial incorporating cetuximab, cisplatin, and irradiation in women with locally advanced cervical cancer is currently ongoing (ClinicalTrials.gov Identifier: NCT00292955).

Angiogenesis is central to cervical cancer development and progression, as it seems to be directly related to HPV inhibition of p53 and subsequent stabilization of hypoxia-inducible factor-one alpha (HIF-1 alpha), both of which increase the levels of vascular endothelial growth factor (VEGF) [59]. Bevacizumab, a monoclonal antibody against VEGF, was recently tested in combination with radiotherapy and cisplatin chemotherapy in a phase II study [46] involving 49 evaluable, previously untreated patients with locally advanced cervical cancer. The study demonstrated the feasibility of the combination, as 76% of the patients received bevacizumab and cisplatin according to the study protocol and 94% of them had both external beam irradiation and brachytherapy administered as per protocol or with acceptable variation [46]. On these grounds, a phase II study of bevacizumab in combination with definitive radiotherapy and cisplatin chemotherapy in the same setting has completed accrual and results are awaited (ClinicalTrials.gov Identifier: NCT00369122). Several VEGF receptor tyrosine kinase inhibitors (VEGFR-TKI), such as sunitinib and pazopanib, are being currently tested in phase II studies in recurrent or metastatic disease (ClinicalTrials.gov Identifier: NCT00389974) but their development in the locally advanced setting is hampered by safety concerns regarding increased rates of fistula formation, especially when combined with external beam irradiation.

## 5. Conclusions

During the past three decades, efforts to enhance cytotoxic activity of radiotherapy using radiosensitizers have evolved from single agent cisplatin administration to sophisticated regimens incorporating various platinum and non-platinum-containing regimens. Most of these regimens led to moderate improvements in response rates and progression-free survival, however, at the cost of moderate to substantial toxicity. Nevertheless, with the sole exception of the cisplatin-gemcitabine combination [33], none of the regimens has thus far been associated with improved survival outcomes. The advent of the bioradiotherapy era is anticipated to lead to the identification of more “sophisticated” therapeutic combinations exploiting the potential synergy between irradiation and molecular inhibition of critical signaling pathways and to enable the design of robust clinical trials based on strong biological rational.

## Figures and Tables

**Table 1:** Randomized phase III clinical trials comparing platinum combinations to platinum monotherapy concurrently with external beam irradiation in patients with locally advanced cervical cancer.

Trial	Year	Experimental regimen	*N*	FIGO stage	Primary outcome	Remarks
Morris et al. [12]	1999	Cisplatin 5-FU	401	IB-IVA	5-year OS rate: 73%	Concurrent CRT decreased the rates for both local and distant recurrence
Rose et al. [11]	1999	Cisplatin 5-FU Hydroxyurea	526	IIB-IVA	5-year OS rate: 64%	Combination more toxic than cisplatin monotherapy
Whitney et al. [10]	1999	Cisplatin 5-FU	388	IIB-IVA	5-year OS rate: 63%	Superior to hydroxyurea alone
Veerasarn et al. [26]	2007	Carboplatin tegafur-uracil	469	IIB-IIIB	5-year PFS and OS were 76% and 93%, respectively	ORR, PFS, and OS not different to carboplatin alone
Kim et al. [28]	2008	Cisplatin 5-FU	158	IIB-IVA	5-year OS rate: 70%	Cisplatin monotherapy equally efficient and less toxic
Lanciano et al. [13]	2005	Cisplatin 5-FU (PVI)	NR	IIB-IVA	1-year PFS and OS were 76% and 93%, respectively	Prematurely terminated for futility compared to cisplatin
Dueñas-González et al. [33]	2011	Cisplatin gemcitabine	515	IIB-IVA	3-year PFS rate: 74.4%	Significant OS benefit compared to cisplatin monotherapy Combination group also received adjuvant chemotherapy

FIGO: International Federation of Gynecologic Oncology; CRT: chemoradiotherapy; OS: overall survival; PFS: progression-free survival; ORR: objective response rate; 5-FU: 5-fluorouracil; PVI: protracted venous infusion; *N*: number of patients.

**Table 2:** Main phase I-II clinical trials of various platinum combinations and non-platinum-containing regimens concurrently with external beam irradiation in patients with locally advanced cervical cancer.

Trial	Year	Phase	Experimental regimen	*N*	FIGO stage	Primary outcome	Remarks
Rao et al. [24]	2005	I	Carboplatin Paclitaxel	15	IB2-IVA	Feasible	2-year PFS and OS were 80% and 86%, respectively
Colombo et al. [27]	1997	II	Carboplatin 5-FU	28	IIA-IVA	5-year OS rate: 66%	ORR, PFS, and OS not different to carboplatin alone
Choi et al. [29]	2008	II	Cisplatin 5-FU	57	IB2-IVA	5-year OS rate: 70%	ORR: 91.5%
Sol et al. [30]	2009	II (randomized)	Cisplatin Paclitaxel	93	IB-IVA	5-year PFS and OS were 79.1% and 80.9%, respectively	Equally efficient but more toxic than cisplatin/5-FU
Domingo et al. [32]	2009	II	Capecitabine	60	IIB-IIIB	ORR: 88%	1-year PFS and OS were 86% and 95%, respectively
Zarbá et al. [34]	2003	I-II	Gemcitabine	36	IIB-IVA	3-year OS rate: 72%	88.8% complete response rate
Zhang et al. [35]	2010	II	Paclitaxel Nedaplatin	34	IIB-IIIB	2-year OS: 93%	88% complete response rate
Geara et al. [36]	2010	II	Paclitaxel	31	IB2-IVA	5-year OS rate: 43%	Inferior to weekly cisplatin
Zanetta et al. [37]	2000	II	Paclitaxel Ifosfamide Cisplatin	38	Bulky locally advanced	ORR: 84.2%	Manageable toxicity
Roberts et al. [38]	2000	II	Mitomycin C	160	IB2-IVA	4-year PFS: 71%	Better than radiotherapy alone
Nguyen et al. [39]	1991	II	Mitomycin C 5-FU	38	IB-IVA	Median OS: 87 months	Significantly higher OS than 5-FU alone
Christie et al. [40]	1995	II	Mitomycin C 5-FU	93	IB-IVA	73.2	4-year survival: 87%
Berclaz et al. [41]	2002	II	Cisplatin Mitomycin C 5-FU	22	IIB-IVA	ORR: 82%	All patients developed acute haematological toxicity
Rose et al. [42]	2012	I	Cisplatin Topotecan	11	IB-IVA	Feasible	Platinum dose reduced to 30 mg/m^2^
Fabbro et al. [43]	2010	I	Cisplatin Irinotecan	15	IB2-IVA	Feasible	2-year survival: 81%

FIGO: International Federation of Gynecologic Oncology; CRT: chemoradiotherapy; OS: overall survival; PFS: progression-free survival; ORR: objective response rate; 5-FU: 5-fluorouracil; PVI: protracted venous infusion; *N*: number of patients.

**Table 3:** Main clinical trials implementing molecular targeted agents concurrently with external beam irradiation in patients with locally advanced cervical cancer.

Trial	Year	Phase	Experimental regimen	*N*	FIGO stage	Primary outcome	Remarks
Nogueira-Rodrigues et al. [44]	2008	I	Erlotinib	15	IIB-IIIB	Feasible	Recommended erlotinib dose: 150 mg
Moore et al. [45]	2012	I	Cetuximab	>20	IIA-IVA	Feasible	Not feasible in patients with extended field radiation therapy
Schefter et al. [46]	2012	I-II	Bevacizumab Cisplatin	60	IB-IIIB	Feasible	Hematologic toxicity common

FIGO: International Federation of Gynecologic Oncology.
